# Bradykinin-induced Ca^2+^ signaling in human subcutaneous fibroblasts involves ATP release via hemichannels leading to P2Y_12_ receptors activation

**DOI:** 10.1186/1478-811X-11-70

**Published:** 2013-09-18

**Authors:** Ana Rita Pinheiro, Diogo Paramos-de-Carvalho, Mariana Certal, Cristina Costa, Maria Teresa Magalhães-Cardoso, Fátima Ferreirinha, Maria Adelina Costa, Paulo Correia-de-Sá

**Affiliations:** 1 Laboratório de Farmacologia e Neurobiologia, Unidade Multidisciplinar de Investigação Biomédica (UMIB), Instituto de Ciências Biomédicas Abel Salazar da Universidade do Porto (ICBAS-UP), Rua Jorge Viterbo Ferreira 228, Edif. 2 Piso 4, Porto 4050-313, Portugal; 2 Área Técnico-Científica de Fisioterapia, Escola Superior de Tecnologia da Saúde do Instituto Politécnico do Porto (ESTSP-IPP), Vila Nova de Gaia, Portugal; 3 Departamento de Química, ICBAS-UP, Porto, Portugal

**Keywords:** ADP, ATP release, Bradykinin, Cx43, Hemichannels, Human subcutaneous fibroblasts, Panx1, P2 purinoceptors

## Abstract

**Background:**

Chronic musculoskeletal pain involves connective tissue remodeling triggered by inflammatory mediators, such as bradykinin. Fibroblast cells signaling involve changes in intracellular Ca^2+^ ([Ca^2+^]_i_). ATP has been related to connective tissue mechanotransduction, remodeling and chronic inflammatory pain, via P2 purinoceptors activation. Here, we investigated the involvement of ATP in bradykinin-induced Ca^2+^ signals in human subcutaneous fibroblasts.

**Results:**

Bradykinin, via B_2_ receptors, caused an abrupt rise in [Ca^2+^]_i_ to a peak that declined to a plateau, which concentration remained constant until washout. The plateau phase was absent in Ca^2+^-free medium; [Ca^2+^]_i_ signal was substantially reduced after depleting intracellular Ca^2+^ stores with thapsigargin. Extracellular ATP inactivation with apyrase decreased the [Ca^2+^]_i_ plateau. Human subcutaneous fibroblasts respond to bradykinin by releasing ATP via connexin and pannexin hemichannels, since blockade of connexins, with 2-octanol or carbenoxolone, and pannexin-1, with ^10^Panx, attenuated bradykinin-induced [Ca^2+^]_i_ plateau, whereas inhibitors of vesicular exocytosis, such as brefeldin A and bafilomycin A1, were inactive. The kinetics of extracellular ATP catabolism favors ADP accumulation in human fibroblast cultures. Inhibition of ectonucleotidase activity and, thus, ADP formation from released ATP with POM-1 or by Mg^2+^ removal from media reduced bradykinin-induced [Ca^2+^]_i_ plateau. Selective blockade of the ADP-sensitive P2Y_12_ receptor with AR-C66096 attenuated bradykinin [Ca^2+^]_i_ plateau, whereas the P2Y_1_ and P2Y_13_ receptor antagonists, respectively MRS 2179 and MRS 2211, were inactive. Human fibroblasts exhibited immunoreactivity against connexin-43, pannexin-1 and P2Y_12_ receptor.

**Conclusions:**

Bradykinin induces ATP release from human subcutaneous fibroblasts via connexin and pannexin-1-containing hemichannels leading to [Ca^2+^]_i_ mobilization through the cooperation of B_2_ and P2Y_12_ receptors.

## Background

Despite its overwhelming size throughout the body, the connective tissue has been generally overlooked or misunderstood. It has been considered as relatively superfluous apart from its supporting role amongst more specialized tissues [1]. It has long been known that scar tissue is a common cause of chronic musculoskeletal pain. Evidences have been produced suggesting that connective tissue may become thicker and less compliant in patients with chronic pain, possible as a result of chronic inflammation and fibrosis [2-4]. Therefore, the normal response to mechanical stretch may be dampened by disturbance of the viscoelastic properties of the subcutaneous connective tissue as a consequence of fibroblast remodeling promoted by inflammatory mediators, like neurotrophins, cytokines, peptides, protons, free radicals, histamine, bradykinin, serotonin, and prostanoids [5]. Since the subcutaneous connective tissue is richly innervated by sensory nerve endings, inputs arising from affected connective tissue may alter pain perception.

Bradykinin is one of the most potent algogenic compounds that is synthesized from inactive precursors, the kininogens, following tissue injury [6] and by contracting skeletal muscles (reviewed in [7]). Bradykinin has been strongly implicated in tissue inflammation [6] and it is also known to be mitogenic in fibroblasts from the human foreskin and lung [8,9]. Bradykinin preferentially induces its physiological effects by binding to the B_2_ receptor subtype. In intact cells, bradykinin was shown to induce the activation of phospholipases A_2_, C and D; the release of prostaglandins, the accumulation of cyclic AMP and of cyclic GMP, and the mobilization of Ca^2+^ were demonstrated (reviewed in [10]). Bradykinin causes a rapid (within 30 s) translocation of protein kinase C isoforms of all groups (classical Ca^2+^-dependent isoform α, new Ca^2+^-independent isoform ϵ, and atypical isoform ζ) from the cytosol to the plasma membrane [10]. Bradykinin-induced translocation of protein kinase C to the plasma membrane may favor enzyme coupling to coexistent extracellular signaling molecules under pathological conditions, thus significantly potentiating their effects.

The way bradykinin is involved in pain perception might involve direct excitation of primary nociceptive afferents and/or the indirect reduction of nociceptors threshold by favoring the release of excitatory signaling mediators [11]. It has been showed that acute bradykinin exposure potentiates algogenic P2X3 purinoceptor-mediated calcium responses from neurons, followed by their down-regulation upon chronic (24 h) exposure. On the other hand, P2Y receptors responses in satellite neuroglia may be upregulated, suggesting a complex interplay between bradykinin and P2 purinoceptors activation in pain pathophysiology [12]. Previous studies demonstrated that bradykinin elicits the release of ATP from various cell types, including smooth muscle fibers, epithelial cells and cardiac endothelial cells from guinea pigs [13], urothelial cells from both human and rats [14,15], and several immortalized cell lines (*e.g.* MDCK, COS-7, HEK-293) (reviewed in [16]). The mechanism of ATP release induced by bradykinin is, however, poorly understood particularly in human tissues. Nucleotides-releasing pathways in intact cells include (1) electrodiffusional translocation via connexin- and pannexin-containing hemichannels and voltage-dependent anion channels, (2) facilitated diffusion by nucleotide-specific ATP-binding cassette (ABC) transporters, and (3) vesicle exocytosis (reviewed in [17]). In parallel to bradykinin, huge amounts of extracellular ATP may leak from damaged cells during mild tissue injury. Once released, ATP may act as an autocrine or paracrine mediator in neighboring cells via ionotropic P2X and metabotropic P2Y purinoceptors activation. ATP signaling may, however, be limited by membrane-bound ectonucleotidases, which sequentially catabolize nucleoside 5’-triphosphates to their respective 5’-di- and monophosphates and adenosine [17]. As a consequence, appearance of ATP and active metabolites, like ADP and adenosine, in the extracellular fluid form concentration gradients enabling differential targeting of subtype-specific purinoceptors and, thus, cell communication and signaling.

Thus, taking into consideration that (1) changes in the regulation of connective tissue ATP signaling may be important in the pathogenesis of chronic inflammatory pain [18] and that (2) algogenic inflammatory mediators, such as bradykinin, may sensitize cells to autocrine and paracrine signals operated by extracellular adenine nucleotides (reviewed in [19]), we investigated the involvement of ATP in bradykinin-induced Ca^2+^ signals in human subcutaneous fibroblasts. Understanding the mechanisms underlying purinergic cell signaling and its interplay with inflammatory mediators in the human subcutaneous connective tissue may highlight new strategies for the treatment of chronic musculoskeletal painful diseases (*e.g.* drug-resistant fibromyalgia).

## Results

### Characterization of human fibroblast cells in culture

**Figure 1:**
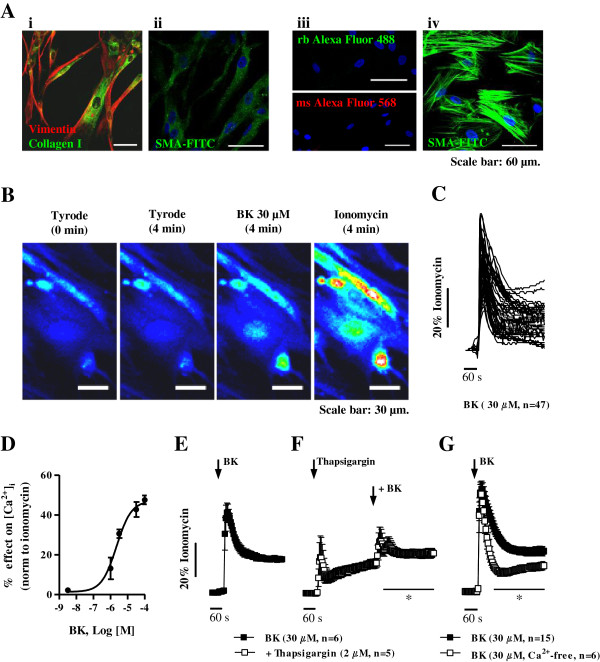
**Bradykinin stimulates the release of intracellular Ca**^**2+ **^**stores and Ca**^**2+ **^**influx from the extracellular space.** Panel **A** shows immunoreactivity of cells cultured from explants of human subcutaneous tissue against fibroblast-cell markers, vimentin (red, **Ai**) and type I collagen (green, **Ai**), and α-smooth muscle actin (SMA-FITC, green, **Aii**). Negative controls, in which cells were incubated only with secondary antibodies, Alexa Fluor 488 (green) and Alexa Fluor 568 (red), are shown for comparison purposes **(Aiii)**; a positive control of SMA-FITC immunoreactivity in rat cardiac myofibroblasts is also shown (green, **Aiv**). Cell nuclei are stained with DAPI (blue); scale bar 60 μm. Panel **B** illustrates intracellular Ca^2+^ ([Ca^2+^]_i_) oscillations in cultured human subcutaneous fibroblasts loaded with the fluorescent calcium indicator, Fluo-4 NW (2.5 μM, see Methods) obtained in the absence and in the presence of bradykinin (BK, 30 μM). Changes in fluorescence were detected in the time-lapse mode with a confocal microscope. Calibration to the maximal calcium load produced by ionomycin (5 μM, 100% response) is also shown for comparison. Image scale bars: 30 μm. Panel **C** shows that the kinetics of BK-induced [Ca^2+^]_i_ signals differed slightly between cells of a given population. Panel **D** depicts the concentration-response curve of [Ca^2+^]_i_ oscillations produced by BK (0.003-100 μM). Panels **E**, **F** and **G**, represent [Ca^2+^]_i_ oscillations produced by BK (30 μM) applied in the absence **(E)** and in the presence of the selective endoplasmic reticulum Ca^2+^-ATPase inhibitor, thapsigargin (2 μM, **F**), and after removal of extracellular Ca^2+^ (Ca^2+^-free medium plus EGTA, 100 μM, **G**). Black arrows indicate the time of drugs application. Each point represents pooled data from an *n* number of experiments. The vertical bars represent S.E.M.. *p < 0.05 represent significant differences from BK (30 μM) alone.

Cultured cells obtained from human subcutaneous connective tissue through the explant technique are elongated and exhibit a spindle-shape morphology, which is characteristic of fibroblasts [20]. At the time that functional experiments were conducted, all cells exhibited positive immunoreactivity against fibroblast-cell markers, vimentin (Figure 1Ai, red) and type I collagen (Figure 1Ai, green) [21], and no specific staining was developed against stress fibers containing α-smooth muscle actin (SMA-FITC, Figure 1Aii). Negative controls, in which cells were incubated only with the secondary antibodies Alexa Fluor 488 (green) and Alexa Fluor 568 (red), are shown in Figure 1Aiii. For comparison purposes, Figure 1Aiv illustrates a positive control of SMA-FITC obtained in rat cardiac myofibroblasts where SMA-immunoreactivity exhibits a clear filamentary pattern (Figure 1Aiv), which was not observed in human subcutaneous fibroblasts (Figure 1Aii).

### Bradykinin, via B_2_ receptors, stimulates the release of intracellular Ca^2+^ stores and Ca^2+^ influx from the extracellular space

Bradykinin (0.001-100 μM) caused prominent intracellular Ca^2+^ ([Ca^2+^]_i_) rises in human subcutaneous fibroblasts (Figure 1). Global changes in [Ca^2+^]_i_ were monitored with a multidetection microplate reader after pre-incubation of the cells with the calcium sensitive dye, Fluo-4 NW; in some instances, single-cell [Ca^2+^]_i_ imaging was also performed using a laser scanning confocal microscope in the time-lapse mode (Figure 1B and 1C) [22]. The effect of bradykinin (0.001-100 μM) was dependent on the concentration (Figure 1D); significant (*p* < 0.05) [Ca^2+^]_i_ rises were observed at concentrations higher than 1 μM. Bradykinin typically produced a biphasic response (Figure 1C); at 30 μM concentration, [Ca^2+^]_i_ raised abruptly to a peak that attained 44 ± 2% of the maximal calcium load produced by ionomycin (5 μM, 100% response), then declined to a plateau of elevated [Ca^2+^]_i_ which remained fairly constant until drug washout (Figure 1E). Bradykinin (30 μM) produced negligible changes in [Ca^2+^]_i_ in the presence of the selective inhibitor of endoplasmic reticulum Ca^2+^-ATPase, thapsigargin (2 μM, n = 5), which is known to deplete intracellular Ca^2+^ stores following a transient (< 2 min) rise of [Ca^2+^]_i_ levels [23] (Figure 1F). Removal of external Ca^2+^ (plus EGTA, 100 μM, n = 6) significantly (*p* < 0.05) attenuated the plateau phase, but the magnitude of the peak was kept almost unchanged (Figure 1G). It, thus, appears that in human subcutaneous fibroblasts the initial transient component must be caused by intracellular Ca^2+^ release from internal stores. The sustained plateau of elevated [Ca^2+^]_i_ results from Ca^2+^ entry through the plasma membrane in response to depletion of Ca^2+^ stores and/or through the concurrent activation of other membrane-bound receptors, namely P2 purinoceptors. This pattern is consistent with previous findings in the literature regarding bradykinin-induced [Ca^2+^]_i_ responses in several cell types [24,25], including human fibroblasts of the foreskin [26].

**Figure 2:**
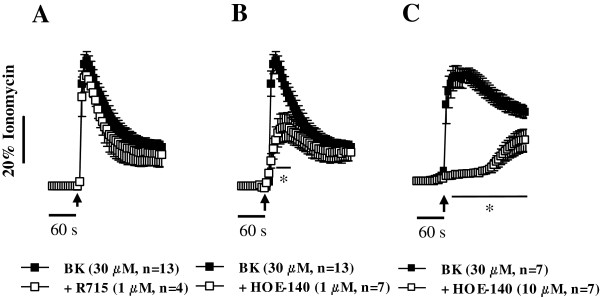
**Bradykinin-induced [Ca**^**2+**^**]**_**i **_**rise in human subcutaneous fibroblasts depends on B**_**2 **_**receptor activation.** Calcium oscillations in human fibroblasts of the subcutaneous tissue stimulated with bradykinin (BK, 30 μM) were tested after pretreatment of the cells with selective B_1_ and B_2_ receptor antagonists, respectively R715 (1 μM, **A**) and HOE-140 (1 μM, **B**; 10 μM, **C**). Cells were pre-incubated with the cell-permeant fluorescent calcium indicator, Fluo-4 NW (2.5 μM, see Methods). Changes in fluorescence were detected using a microplate reader. Intracellular Ca^2+^ transients were calibrated to the maximal calcium load produced by ionomycin (5 μM, 100% response). Black arrows indicate the time of drugs application. No changes in baseline fluorescence were observed after application of the antagonists. Each point represents pooled data from an *n* number of experiments. The vertical bars represent S.E.M.. *p < 0.05 represent significant differences from BK (30 μM) alone.

Bradykinin-induced [Ca^2+^]_i_ rise in human subcutaneous fibroblasts was concentration-dependently attenuated by the selective B_2_ receptor antagonist, HOE-140 (1 and 10 μM, n = 7) (Figure 2B and 2C), whereas selective blockade of the B_1_ receptor with R715 (1 μM, n = 4) was without effect (Figure 2A). At the highest concentration (10 μM), HOE-140 significantly (*p* < 0.05) decreased, but did not completely block, the late phase of bradykinin-induced [Ca^2+^]_i_ response given that the peptide was used in a concentration (30 μM) near that necessary for saturation of B_2_ receptors in these cells (see Figure 1D). On their own, the two antagonists were devoid of any significant effect.

### Bradykinin induces ATP release from human subcutaneous fibroblasts: involvement of intracellular Ca^2+^ stores

**Figure 3:**
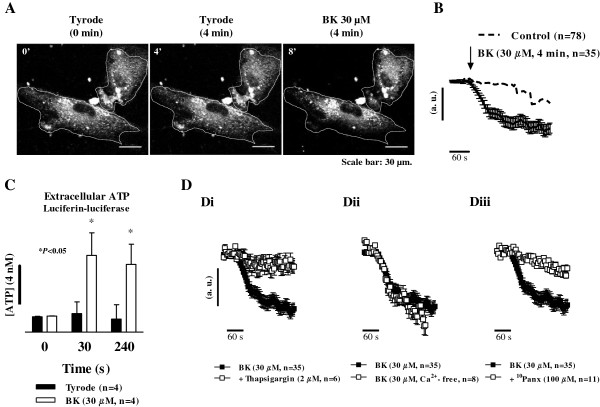
**Bradykinin elicits ATP release from human fibroblasts by a mechanism depending on intracellular Ca**^**2+ **^**mobilization.** Panels **A**, **B** and **D** represent cells loaded with quinacrine (30 μM, a fluorescent dye that specifically binds ATP), for 60 min at 37°C. ATP release was detected by single-cell confocal microscopy in the time-lapse mode measuring the fluorescence intensity decay 4 min after bradykinin (BK, 30 μM, **A** and **B**) application as compared to the control situation, in which only Tyrode’s solution was applied **(A)**. Panel **D**, shows the effect of BK (30 μM) after pretreatment of the cells with the selective endoplasmic reticulum Ca^2+^-ATPase inhibitor, thapsigargin (2 μM, **Di**), and after removal of extracellular Ca^2+^ (Ca^2+^-free medium plus EGTA, 100 μM, **Dii**); the effect of BK (30 μM) in the presence of the selective Panx1 inhibitor, ^10^Panx (100 μM, **Diii**), is also shown. Image scale bars: 30 μm. Graphs show quinacrine fluorescence decay (arbitrary units, a.u.) plotted versus time in the presence of Tyrode’s solution **(B)**, thapsigargin (2 μM, **Di**) and Ca^2+^-free medium **(Dii)**. Black arrows indicate the time of drugs application. Each point represents pooled data from an *n* number of cells. The vertical bars represent S.E.M.. Panel **C**, shows the ATP content in human subcutaneous fibroblast cultures at given time intervals (0-240 seconds) in the presence of BK (30 μM, open bars) as compared to the control condition where only Tyrode’s solution was applied (closed bars). Relative luminescence units (RLUs) were calibrated using a 4 nM ATP standard (left hand-side black vertical bar). Each bar represents pooled data from an *n* number of experiments. The vertical bars represent S.E.M.. *p < 0.05 represent significant differences from BK (30 μM) alone.

The release of ATP from human subcutaneous fibroblasts in culture was inferred from destaining of cells loaded with quinacrine, an ATP-binding intracellular fluorescent dye, by confocal microscopy in the time-lapse mode. Bradykinin (30 μM, n = 35) increased (*p* < 0.05) fluorescence intensity decay of cells loaded with quinacrine as compared to the control situation in which the cells were challenged with the Tyrode’s solution (Figure 3A and 3B). Quinacrine destaining of cells exposed to bradykinin was more evident at the periphery than near the nucleus (Figure 3A). Confirmation that human subcutaneous fibroblasts release ATP in response to bradykinin (30 μM, n = 4) was obtained by measuring the luminescence of the medium before and after bradykinin application to cells incubated with luciferin-luciferase (Figure 3C). Results demonstrate that ATP release peaked at 30 s after bradykinin (30 μM) application and was kept fairly constant during the 4-min drug application.

Pretreatment with thapsigargin (2 μM, n = 6) significantly (*p* < 0.05) attenuated bradykinin-induced quinacrine destaining (Figure 3Di). In contrast, removal of extracellular Ca^2+^ (plus EGTA, 100 μM, n = 8) from the incubation medium did not significantly (*p* > 0.05) affect the fluorescence intensity decay of quinacrine stained ATP granules (Figure 3Dii). These observations indicate that Ca^2+^ recruitment from thapsigargin-sensitive internal stores is required for the release of ATP induced by bradykinin.

### ATP release via connexin and pannexin-1 hemichannels contributes to bradykinin-induced [Ca^2+^]_i_ mobilization

**Figure 4:**
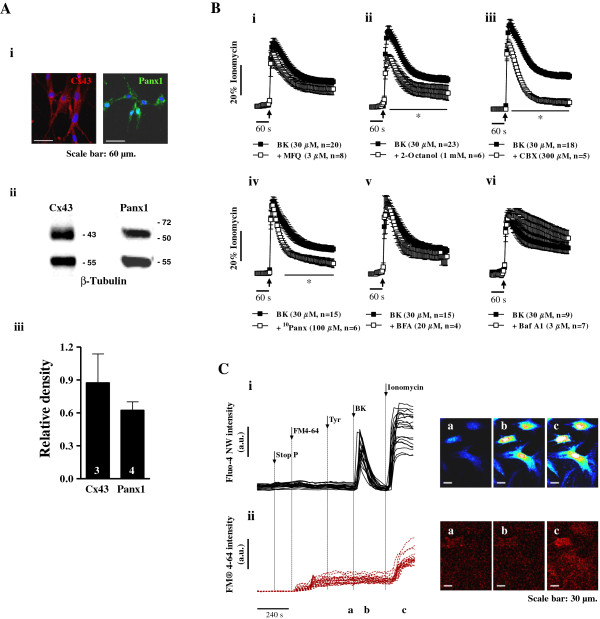
**Bradykinin-induced [Ca**^**2+**^**]**_**i **_**signals in human subcutaneous fibroblasts involve connexin and pannexin-1 containing hemichannels opening.** Panel **A** shows representative confocal micrographs **(Ai)** and blots **(Aii)** of Cx43 and Panx1 hemichannels immunoreactivity in cultured human subcutaneous fibroblasts from 3-4 individuals. Image scale bars: 60 μm. β-tubulin was used as a reference protein to determine the relative density of Cx43 and Panx1 immunoblots **(Aiii)**. Panel **B** shows the effect of bradykinin (BK, 30 μM) in the presence of inhibitors of connexin and/or Panx1 hemichannels, namely mefloquine (MFQ, 3 μM, **Bi**), 2-octanol (1 mM, **Bii**), carbenoxolone (CBX 300 μM, **Biii**) and ^10^Panx (100 μM, **Biv**). The effects of the vesicular transport inhibitor, brefeldin A (BFA, **Bv**) and of the specific inhibitor of vacuolar H^+^-ATPases, bafilomycin A1 (Baf A1, **Bvi**), are also shown for comparison. Cells were pre-incubated with the cell-permeant fluorescent calcium indicator, Fluo-4 NW (2.5 μM, see Methods). Changes in fluorescence were detected using a microplate reader. [Ca^2+^]_i_ transients were calibrated to the maximal calcium load produced by ionomycin (5 μM, 100% response). Black arrows indicate the time of drugs application. None of the inhibitors significantly change baseline fluorescence when applied alone. Each point represents pooled data from an *n* number of experiments. The vertical bars represent S.E.M.. *p < 0.05 represent significant differences from BK (30 μM) alone. Panel **C** shows representative traces of single-cell [Ca^2+^]_i_ oscillations in rat subcutaneous fibroblasts loaded with Fluo-4 NW **(Ci)** obtained in parallel with the incorporation of the membrane-selective fluorescent dye, FM4-64 **(Cii)**, used to evaluate vesicle endocytosis and exocytosis in living cells. Changes in fluorescence were detected in the time-lapse mode using a laser-scanning confocal microscope. Vertical lines indicate the time of drugs application. Fluorescence confocal micrographs were obtained at the indicated time points (a, b and c). Image scale bars: 30 μm.

Among other mechanisms, hemichannels containing connexins (Cx) and pannexin-1 (Panx1) are now widely accepted as putative mediators of ATP translocation to the extracellular milieu in non-excitable cells. The expression of Cx43 is characteristic of fibroblasts from multiple tissue origins [27,28]. Using immunofluorescence confocal microscopy (Figure 4Ai) and Western blot analysis (Figure 4Aii), we demonstrated that fibroblasts of the human subcutaneous tissue in culture express anti-Panx1 (~50 kDa) immunoreactivity in addition to the expected Cx43 (43 kDa). Panx1 and Cx43 are both expressed in relatively high density as compared to β-tubulin standard protein levels (Figure 4Aiii).

These findings prompted us to test whether bradykinin-induced [Ca^2+^]_i_ oscillations in human subcutaneous fibroblasts depend on the release of ATP via hemichannels using subtype selective connexin and pannexin-1 inhibitors. Inhibition of Cx36- and Cx50-containing hemichannels with mefloquine (MFQ, 3 μM, n = 8) [29,30] was devoid of effect on bradykinin-induced [Ca^2+^]_i_ rise (Figure 4Bi). On the other hand, 2-octanol (1 mM, n = 6), which blocks Cx43, Cx46 and Cx50 hemichannels [30,31] (Figure 4Bii), and carbenoxolone (CBX, 300 μM, n = 5), a non-selective inhibitor of connexins Cx26, Cx30, Cx32, Cx43 and Cx46, which also blocks Panx1-containing hemichannels [31] (Figure 4Biii), significantly (*p* < 0.05) attenuated [Ca^2+^]_i_ response induced by bradykinin (30 μM). Interestingly, the selective Panx1 mimetic inhibitory peptide, ^10^Panx (100 μM, n = 6) [32], also decreased the bradykinin-induced [Ca^2+^]_i_ response (Figure 4Biv). CBX (300 μM) was the most effective of the three inhibitors, probably because it has a broad inhibitory spectrum blocking equally well connexin and Panx1 containing hemichannels. Coincidently or not, 2-octanol (1 mM), CBX (300 μM) and ^10^Panx (100 μM), were more effective in depressing the plateau phase of bradykinin (30 μM) response (Figures 4Bii-4Biv), although 2-octanol (1 mM) and CBX (300 μM) also inhibited (*p* < 0.05) the fast [Ca^2+^]_i_ rise induced by the peptide. Confocal microscopy studies demonstrated that ^10^Panx (100 μM) also attenuated (*p* < 0.05) bradykinin (30 μM)-induced ATP release from human fibroblasts loaded with quinacrine (see Figure 3Diii).

In order to test if bradykinin-induced [Ca^2+^]_i_ oscillations involved nucleotides-release by exocytosis we used the vesicular transport inhibitor, brefeldin A (BFA, Figure 4Bv), and the specific inhibitor of H^+^-ATPases of the vacuolar type, bafilomycin A1 (Baf A1, Figure 4Bvi). No statistical significant (*p* > 0.05) differences were found in [Ca^2+^]_i_ oscillations produced by bradykinin (30 μM) in the absence and in the presence of BFA (20 μM, n = 4) and Baf A1 (3 μM, n = 7). Confocal microscopy experiments with primary cultures of rat subcutaneous fibroblasts loaded with the calcium sensitive dye, Fluo-4 NW, and then incubated with FM4-64, a membrane-selective fluorescent dye, were used to evaluate vesicle endocytosis and exocytosis in the time lapse mode [33,34]. Results depicted in Figure 4Ci, show that bradykinin (30 μM)-induced [Ca^2+^]_i_ oscillations were not accompanied by measurable changes in FM4-64 fluorescence signals in the same cells (Figure 4Cii). Ionomycin (5 μM) increased moderately FM4-64 fluorescence labeling because plasma membrane modifications (e.g. membrane protrusions, microvesiculation, blebbing) are likely to occur in the presence of the Ca^2+^ ionophore. Overall, our findings suggest that ATP release via connexins (most probable Cx43) and Panx1 hemichannels, rather than by vesicle exocytosis, may contribute importantly to the plateau phase of bradykinin-induced [Ca^2+^]_i_ response in fibroblasts cultured from the human subcutaneous connective tissue.

### The plateau phase of bradykinin [Ca^2+^]_i_ recruitment is partially dependent on the activation of ADP-sensitive P2Y_12_ purinoceptors

**Figure 5:**
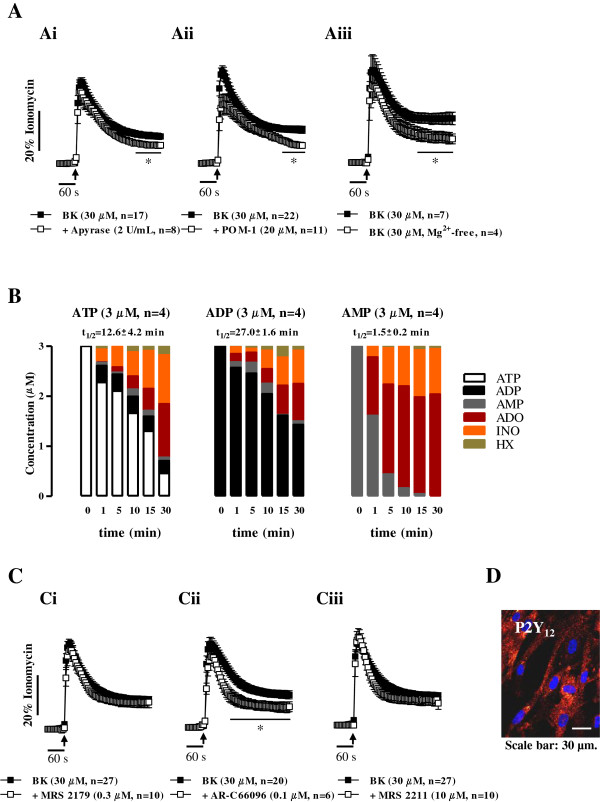
**Bradykinin-induced [Ca**^**2+**^**]**_**i **_**mobilization is partially dependent on the activation of P2Y**_**12 **_**purinoceptors.** Panel **A** shows the effect of bradykinin (BK, 30 μM) after pretreating human subcutaneous fibroblasts with apyrase (2 U/mL, **Ai**), which catabolizes ATP/ADP into AMP, and after inhibition of ectonucleotidases, with POM-1 (20 μM, **Aii**) or after removing Mg^2+^ from the incubation fluid **(Aiii)**. Panel **C** shows the effects of BK (30 μM) in the absence or presence of selective P2Y_1_, P2Y_12_ and P2Y_13_ receptor antagonists, respectively MRS 2179 (0.3 μM, **Ci**), AR-C 66096 (0.1 μM, **Cii**) and MRS 2211 (10 μM, **Ciii**). Cells were pre-incubated with the cell-permeant fluorescent calcium indicator, Fluo-4 NW (2.5 μM, see Methods). [Ca^2+^]_i_ transients were calibrated to the maximal calcium load produced by ionomycin (5 μM, 100% response). Black arrows indicate the time of drugs application. No changes in baseline fluorescence were observed after application of the modulators. Each point represents pooled data from an *n* number of experiments. The vertical bars represent S.E.M.. *p < 0.05 represent significant differences from BK (30 μM) alone. Panel **B** illustrates the time course of the extracellular catabolism of adenine nucleotides in human subcutaneous fibroblasts grown in culture for 11 days. ATP, ADP or AMP (3 μM) were added to the culture medium at time zero; samples (75 μl) were collected at indicated times. Each sample was analyzed by HPLC to separate and quantify ATP (white), ADP (black), AMP (grey), adenosine (ADO, red), inosine (INO, orange) and hypoxanthine (HX, green). Each point represents pooled data from two individuals; 2 replicas were performed for each individual. The calculated half-life time (t_½_) for each initial substrate is shown for comparison. Panel **D** shows immunoreactivity of human subcutaneous fibroblasts against the P2Y_12_ receptor; shown image is representative of three independent experiments. Image scale bar is 30 μm.

Extracellular inactivation of ATP directly into AMP with apyrase (NTPDase1 or CD39, 2 U/mL, n = 8) reduced significantly (*p* < 0.05) the plateau phase of bradykinin (30 μM, n = 17) response, while keeping fairly conserved the magnitude of the initial [Ca^2+^]_i_ rise (Figure 5Ai). Surprisingly, inhibition of membrane-bound NTPDases with POM-1 (20 μM, n = 11) [35] also decreased the plateau phase of bradykinin-induced [Ca^2+^]_i_ response in human subcutaneous fibroblasts (Figure 5Aii). The result obtained with POM-1 (20 μM) was confirmed when we tested the effect of bradykinin on [Ca^2+^]_i_ oscillations in the absence of Mg^2+^, an ion that must be present in millimolar concentration in the extracellular fluid for maximum activity of ectonucleotidases [17] (Figure 5Aiii). These findings provide the first evidence that the plateau phase of bradykinin-induced [Ca^2+^]_i_ accumulation by human subcutaneous fibroblasts requires the release of ATP and its subsequent conversion into other biologically active metabolites, most probably ADP, by ectonucleotidases.

The kinetics of the extracellular catabolism of adenine nucleotides (ATP, ADP and AMP) and formation of metabolites in fibroblasts cultured from the human subcutaneous tissue is shown in Figure 5B. Average half-lives of ATP, ADP and AMP were respectively 12.6 ± 4.2 min, 27.0 ± 1.6 min and 1.5 ± 0.2 min (n = 4 observations from two individuals), when the substrates were used in a 3 μM concentration. ATP (3 μM) was sequentially metabolized into ADP, adenosine (ADO), inosine (INO) and hypoxanthine (HX) (Figure 5B). AMP (3 μM) was rapidly and sequentially converted into ADO and INO respectively by ecto-5’-nucleotidase (CD73) and adenosine deaminase (ADA), which might explain why AMP accumulation was almost negligible when ATP (3 μM) and ADP (3 μM) were used as substrates.

The analysis of the corresponding half-life time values clearly indicates that the extracellular catabolism of ADP into AMP is the rate-limiting step to generate ADO from exogenously added adenine nucleotides in cultured human subcutaneous fibroblasts. Therefore, transient accumulation of ADP in the cultures is in favor of a preferential activation of ADP-sensitive P2Y purinoceptors. To investigate the contribution of ADP-sensitive P2Y purinoceptors activation to bradykinin (30 μM)-induced [Ca^2+^]_i_ response in human subcutaneous fibroblasts, we tested its effect in the presence of selective P2Y_1_, P2Y_12_ and P2Y_13_ receptors antagonists (Figure 5C). Selective blockade of the P2Y_12_ receptor with AR-C 66096 (0.1 μM, n = 6) significantly (*p* < 0.05) attenuated the plateau phase of [Ca^2+^]_i_ rise caused by bradykinin (30 μM, n = 20) without much affecting the magnitude of the initial [Ca^2+^]_i_ rise (Figure 5Cii). No significant differences (*p* > 0.05) were observed in the presence of MRS 2179 (0.3 μM, n = 10) and MRS 2211 (10 μM, n = 10) which selectively antagonize P2Y_1_ and P2Y_13_ receptors, respectively (Figure 5Ci and 5Ciii). None of these antagonists modified *per se* [Ca^2+^]_i_ in human subcutaneous fibroblasts. The expression of the P2Y_12_ receptor in cultured human subcutaneous fibroblasts was confirmed by immunocytochemistry (Figure 5D).

### Adenine nucleotides, but not adenosine, increase the influx of Ca^2+^ in human subcutaneous fibroblasts

**Figure 6:**
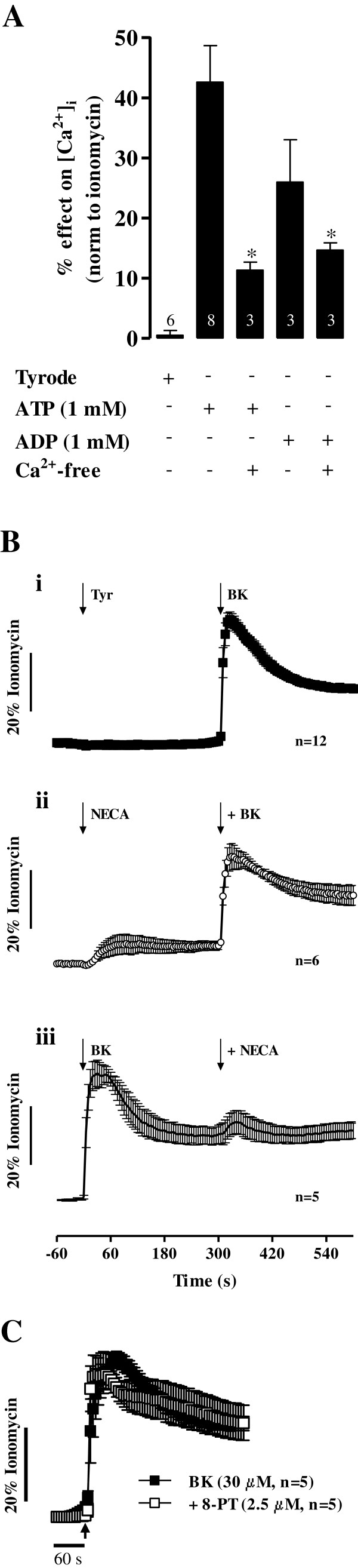
**Adenine nucleotides, but not adenosine, increase Ca**^**2+ **^**influx from the extracellular space in human subcutaneous fibroblasts.** In panel **A**, human subcutaneous fibroblasts were incubated with 1 mM ATP or ADP in control conditions and in Ca^2+^-free medium (plus EGTA, 100 μM). Each bar represents pooled from an *n* number of experiments. The vertical bars represent S.E.M.. *p < 0.05 represent significant differences compared with the effect of ATP or ADP alone. Panel **B** illustrates [Ca^2+^]_i_ oscillations caused by 5’-(N-ethylcarboxamide) adenosine (NECA, 10 μM), an enzymatically stable non-selective adenosine receptor agonist, compared with the effect of bradykinin (BK, 30 μM) in fibroblasts of the human subcutaneous tissue **(Bi)**. NECA (10 μM) and BK (30 μM) were applied cumulatively in sequence **(Bii)** and in the reverse mode **(Biii)**. Panel **C** shows BK (30 μM)-induced [Ca^2+^]_i_ oscillations in the absence and in the presence of a non-selective adenosine receptor antagonist, 8-phenyltheophylline (8-PT, 2.5 μM). Cells were pre-incubated with the cell-permeant fluorescent calcium indicator, Fluo-4 NW (2.5 μM, see Methods). Changes in fluorescence were detected using a microplate reader. [Ca^2+^]_i_ transients were calibrated to the maximal calcium load produced by ionomycin (5 μM, 100% response). Black arrows indicate the time of drugs application. No changes in baseline fluorescence was observed after application of 8-PT (2.5 μM). Each point represents pooled data from an *n* number of experiments. The vertical bars represent S.E.M..

Changes in [Ca^2+^]_i_ in human subcutaneous fibroblasts treated with adenine nucleotides (1 mM) in the presence and in the absence of extracellular Ca^2+^ are shown in Figure 6A. Exogenous application of ATP and ADP (1 mM) transiently increased intracellular [Ca^2+^]_i_ reaching, respectively, 43 ± 4% (n = 8) and 25 ± 8% (n = 3) of the maximal calcium load caused by ionomycin (5 μM, 100% response) within 20 sec from drug application. In Ca^2+^-free medium (plus EGTA, 100 μM), ATP- and ADP-induced [Ca^2+^]_i_ transients were partially but significantly (*p* < 0.05) attenuated (Figure 6A).

Adenosine (ADO) is a recognized anti-nociceptive agent (see *e.g.*[36]) and plays prominent roles in fibroblasts proliferation and tissue remodeling in the skin, liver and lung (reviewed in [37]), yet contradictory effects of the nucleoside have been described on cardiac fibroblasts ([38,39]). Given the high amounts of ADO accumulating in cultured fibroblasts of the human subcutaneous tissue as a result of the extracellular catabolism of released adenine nucleotides (see Figure 5), we sought to investigate the contribution of the nucleoside to bradykinin-induced Ca^2+^ signals in these cells. To this end, we used the enzymatically stable adenosine analogue, 5’-(N-ethylcarboxamide) adenosine (NECA), which non-selectively activates all four adenosine receptor subtypes in the submicromolar range. NECA (10 μM, n = 6) had a negligible effect on [Ca^2+^]_i_ in human subcutaneous fibroblasts when it was applied either alone (Figure 6Bii) or in the presence of bradykinin (30 μM, n = 5) (Figure 6Biii). No statistical significant (*p* > 0.05) difference was found in [Ca^2+^]_i_ oscillations caused by bradykinin (30 μM) in the presence of NECA (10 μM, n = 6) (Figure 6Bii) as compared to the control situation (Figure 6Bi). Moreover, pretreatment of the cells with the non-selective adenosine receptor antagonist, 8-phenyltheophylline (8-PT, 2.5 μM, n = 5), was devoid of effect on bradykinin-induced [Ca^2+^]_i_ rise (Figure 6C), suggesting that adenosine has a minor effect on Ca^2+^ signaling operated by bradykinin in human subcutaneous fibroblasts.

## Discussion

Bradykinin is produced within the interstitium of most tissues and plays an important role in normal and pathological conditions, being considered an important inflammatory pain mediator (reviewed in [40]) that is associated with chronic musculoskeletal pain syndromes [41,42]. Recent findings have shown that in the central nervous system bradykinin triggers astrocyte-neuron signaling via glutamate release followed by NMDA receptors activation [43]. In peripheral tissues, bradykinin receptors have already been described in sensory neurons of dorsal root ganglia (DRG), where it exerts direct and indirect effects via neuronal excitation and threshold modulation by the release of excitatory signaling molecules, respectively [11]. To the best of our knowledge, this is the first report demonstrating that fibroblasts isolated from the human subcutaneous connective tissue respond to bradykinin by releasing ATP into the extracellular medium through the activation of B_2_ receptors, which are constitutively expressed in most of the tissues exhibiting bradykinin sensitivity. Although our experiments were conducted in non-stressful conditions, the involvement of inducible B_1_ receptors mediating bradykinin effects in human subcutaneous fibroblasts cannot be ruled out [44]. Bradykinin-induced ATP release from these cells was demonstrated by two distinct experimental approaches: the luciferin-luciferase bioluminescence assay and ATP-binding quinacrine dye destaining by time-lapse confocal microscopy. Our data also suggest that bradykinin-induced ATP release from human subcutaneous fibroblasts requires Ca^2+^ recruitment from thapsigargin-sensitive internal stores and occurs independently of extracellular Ca^2+^ by electrodiffusional translocation of the nucleotide via hemichannels containing connexins (most probably Cx43) and Panx1.

In several cell types [24,25], including human fibroblasts of the foreskin [26], bradykinin elicits an abrupt rise in [Ca^2+^]_i_ to a peak that depends on Ca^2+^ recruitment from internal stores, which then declines to a plateau that is sustained by Ca^2+^ entry from the extracellular compartment. The long lasting elevation of [Ca^2+^]_i_ seen after the initial [Ca^2+^]_i_ peak probably results from a receptor- or second messenger-operated Ca^2+^ current activated by bradykinin, rather than via a capacitative pathway [26]. Here, we demonstrate for the first time that ADP-sensitive P2Y_12_ purinoceptors may be, at least in part, responsible for the Ca^2+^ influx that is characteristic of the plateau phase of elevated [Ca^2+^]_i_ caused by bradykinin in human subcutaneous fibroblasts. This conclusion is supported by results showing that: (1) human subcutaneous fibroblasts exhibit P2Y_12_ receptor immunoreactivity, (2) bradykinin induces the release of ATP that is subsequently hydrolyzed into ADP by membrane-bound ectonucleotidases, (3) ADP has a tendency to accumulate transiently in the cultures, given that we found that the extracellular catabolism of ADP into AMP is the rate-limiting step of the ectonucleotidase cascade, and, finally, (4) adenine nucleotides-induced [Ca^2+^]_i_ rises in human subcutaneous fibroblasts are dependent on extracellular Ca^2+^.

The pathways underlying bradykinin-induced long lasting Ca^2+^ influx resulting from the cooperation with ADP-sensitive P2Y_12_ purinoceptors need to be further investigated. Interestingly, bradykinin causes a rapid translocation of all protein kinase C isoforms from the cytosol to the plasma membrane within the time frame (30 s) of ATP appearance in the cultures [10], which may favor the synergism between B_2_ and P2Y_12_ receptors activation. The involvement of ADP-sensitive P2Y_12_ receptors in neuropathic pain, has been already reported; authors suggested that this could be due to an unclear mechanism involving non-neuronal cells, such as spinal microglia [45,46] and trigeminal ganglion satellite cells [47]. The close proximity between P2Y_12_ receptor-expressing fibroblasts of the subcutaneous tissue and sensory nerve fibers exhibiting numerous ligand-gated receptors (including ionotropic P2X and metabotropic P2Y purinoceptors) implies that adenine nucleotides released from stimulated fibroblasts may alter acute and chronic pain perception. During acute tissue injury, excessive ATP release from damaged fibroblasts, keratinocytes, blood vessels and inflammatory cells may cause pain by activating excitatory purinoceptors on nociceptive sensory nerve endings [46,48-50]. Lower levels of ATP released from intact cells in response to mild mechanical and thermal stimulation may participate in normal tactile sensation and also contribute to the spontaneous pain and tactile hypersensitivity that occurs under chronic painful conditions involving the subcutaneous connective tissue when nerve endings become sensitized [48,51]. We, now, postulated a new type of interaction involving an autocrine action of ATP, via P2Y_12_ receptors, triggered by the activation of bradykinin B_2_ receptors in human subcutaneous fibroblasts. Moreover, increased sensitivity to extracellular ATP has been described in fibroblasts from patients affected with systemic sclerosis [52].

Little is known about the mechanisms upstream the nucleotide release from human fibroblasts despite the importance of connective tissue ATP signaling in the pathogenesis of acute and chronic inflammatory pain [18,19]. Multiple nucleotide-releasing pathways have been identified in intact cells, which represent a critical component for the initiation of the purinergic signaling cascade (reviewed by [19]). Experiments designed to manipulate exocytosis of vesicles/organelles containing compartmentalized ATP suggest that it might not represent an important pathway for releasing ATP from human fibroblasts stimulated with bradykinin. Several non-vesicular ATP release mechanisms have been proposed, yet many remain controversial and are complicated by the non-specificity of available inhibitors. Hemichannels possessing connexin and Panx1 subunits represent an important mechanism for the cellular release of ATP. The opening of hemichannels occurs in response to many physiological and pathological situations, including volume regulation, proliferation, calcium wave propagation by extracellular messengers and cell death during metabolic inhibition (reviewed in [53]). Using immunofluorescence confocal microscopy and Western blot analysis, we demonstrated that fibroblasts of the human subcutaneous tissue exhibit strong anti-Panx1 immunoreactivity in addition to Cx43 that is characteristic from fibroblasts of other tissue origins [27,28]. Moreover, functional data using non-selective connexin inhibitors targeting Cx43 hemichannels (*e.g.* 2-octanol, CBX) strongly depressed the plateau phase of bradykinin-induced [Ca^2+^]_i_ response. Because connexin hemichannels are activated by moderate (< 500 nM) [Ca^2+^]_i_ elevations, these channels may open in response to bradykinin during the initial [Ca^2+^]_i_ rise [54] and contribute to ATP release and to the subsequent purinoceptor-mediated signaling. Closing of connexin containing hemichannels, which unlike Panx1 hemichannels seal when the spike amplitude rises above 500 nM, contributes to shape bradykinin-induced [Ca^2+^]_i_ oscillations as demonstrated by the partial reduction of the initial [Ca^2+^]_i_ rise of bradykinin in the presence of 2-octanol and CBX.

Considering the relatively high potency of CBX (300 μM) and the fact that this compound also blocks Panx1 containing hemichannels [31], we tested the effect of the selective Panx1 mimetic inhibitory peptide, ^10^Panx, which also depressed the plateau phase of the bradykinin-induced [Ca^2+^]_i_ response in parallel to the decline of ATP release from fibroblasts loaded with quinacrine. Like the effects obtained with apyrase and with the selective P2Y_12_ antagonist, AR-C 66096, inhibition of hemichannels containing Cx43 and Panx1 were more effective in depressing the plateau phase rather than the peak of the bradykinin response. Taken together, data suggest that Cx43 and Panx1 containing hemichannels have a predominant role on ATP release from human subcutaneous fibroblasts stimulated with bradykinin, thereby instigating the regenerative propagation of intracellular Ca^2+^ signals. Our findings agree with the observation that mechanically stimulated cardiac fibroblasts release ATP in a CBX-sensitive manner, an effect that the authors attributed to Cx43 hemichannels not excluding a possible involvement of Panx1 containing hemichannels [55].

Regardless of whether channel-mediated efflux or vesicle exocytosis comprises the predominant ATP release mechanism, most studies (but not all, see *e.g.*[56]) have identified elevation of cytosolic Ca^2+^ as an important regulator of nucleotide export in different cell model systems. The molecular mechanism by which bradykinin releases ATP through the opening of Cx43 and Panx1 hemichannels in human subcutaneous fibroblasts may be the generation of inositol trisphosphate (IP_3_) by phospholipase C and the downstream [Ca^2+^]_i_ mobilization from internal stores [57,58]. Our data, showing that intracellular Ca^2+^ depletion with thapsigargin impaired quinacrine dye destaining induced by bradykinin is in favor with the hypothesis that Ca^2+^ mobilization is necessary for ATP release in these cells. Further experiments are required to discard the ability of bradykinin, like certain G_q_-coupled receptors, to additionally stimulate Rho-GTPase acting to strongly potentiate a Ca^2+^-activated ATP release pathway [59,60]. Seminario-Vidal and col. (2009) [60], demonstrated that Ca^2+^- and RhoA/Rho kinase-dependent ATP release from thrombin-stimulated A549 lung epithelial cells occurs via connexin or pannexin hemichannels, a pathway that seems to be not competent for ATP release in human astrocytoma cells [59]. Given the actions exerted by Rho/Rho kinase on cytoskeleton components (*e.g.* regulating myosin-light chain phosphorylation and actin polymerization), those authors speculated that Rho-promoted membrane-cytoskeletal rearrangements facilitate the insertion of hemichannel subunits within the plasma membrane.

Bradykinin can increase glutamate release from mouse astrocytes through volume-sensitive outwardly rectifying (VSOR) anion channels without cell swelling via a mechanism that is regulated by high intracellular Ca^2+^ in nanodomains [61]. Although we did not test directly whether this pathway plays a role in bradykinin-induced [Ca^2+^]_i_-dependent ATP release from human subcutaneous fibroblasts, it appears that VSOR currents would exhibit a slow activation kinetics requiring 15-20 min after bradykinin application to reach a sustained plateau [61]. This activation pattern is entirely different from the rapid (within 30 s) ATP releasing response to bradykinin observed in human fibroblasts (see Figure 3), thus indicating that slow activating but prolonged VSOR currents play a minor, if some, role in the release of ATP in our experimental time frame. In our hands mefloquine (MFQ, 3 μM, Figure 4Bi), which also blocks non-selectively anion channels, failed to modify bradykinin-induced [Ca^2+^]_i_ signals. Moreover, modulation of VSOR channel permeability through the activation of protein kinase C with the phorbol ester (12-myristate 13-acetate, 1-10 μM, n = 3) did not mimic the effect of bradykinin.

Involvement of soluble signaling mediators, such as ATP and/or its metabolites, may also explain heterogeneity of individual [Ca^2+^]_i_ responses to bradykinin within a cell population; confocal microscopy studies showed that some cells displayed no plateau phase whereas others were not noticeably affected by bradykinin removal and continued to respond for a few minutes (see Figure 1C). This is consistent with the generation of concentration gradients by released ATP and metabolites formation (namely ADP) via ectonucleotidases, which enables differential targeting of subtype-specific P2 purinoceptors and, thus, cell-to-cell communication depending on proximity. Therefore, we may speculate that bradykinin-stimulated fibroblasts trigger a “purinergic wave” mediated by released ATP and metabolites formation that can affect sensory afferent nerve endings localized in the vicinity, representing the first insights of a fibroblast-neuron communication unproved so far.

Recently, it has been reported that mechanical stimulation of human epidermal keratinocytes induces propagating Ca^2+^ waves depending on non-vesicular release of ATP through connexin hemichannels [48]. In view of the potential contribution of the cutaneous release of ATP to acute and chronic pain syndromes, this and other groups demonstrated that human epidermal keratinocytes co-cultured with neurons of the dorsal root ganglia interplay through the release of ATP following keratinocytes-born [Ca^2+^]_i_ waves [62]. Likewise, subcutaneous inflammation or injection of ATP causes pain sensation through the activation of P2X3 receptors expressed in sensory nerve endings, which may become sensitized in both animal models and human patients [63,64]. Knocking down or selectively antagonizing P2X3 receptor activity results in reduced responses to ATP, as well as reduced thermal and mechanical hyperalgesia in inflammatory and neuropathic pain rat models [65,66]. P2Y purinoceptors, especially P2Y_1_ and P2Y_2_, expressed in primary sensory endings have also been implicated in chronic pain states [62]. Authors from the latter study agree that cutaneous ATP release does not appear to contribute to pain sensation in the absence of tissue injury. However, under chronic painful conditions, such as inflammation and nerve injury, nerve endings may become sensitized and a normally innocuous level of subcutaneous ATP may now be sufficient to reach the firing threshold of nociceptors [67]. Despite direct modulation of nociceptors threshold by ATP released from different cell types may play a key role to the association between subcutaneous connective tissue injury and musculoskeletal pain, there are alternative mechanisms that should also be considered in this context. These include interference with muscle tension due to fusimotor effects induced by group III and IV afferents activation, which by projecting to γ-motoneurons amplify muscle spindles activity and, thereby, increases muscle tone, generating metabolites (*e.g.* bradykinin) that enter in a positive feedback loop (reviewed by [41,42,68]).

In addition, it has been proposed that mechanical deformation of the skin by needles and application of heat or electrical current leads to release of large amounts of ATP from keratinocytes, fibroblasts and other cells in skin [46]. Impulses generated by P2 purinoceptors in sensory fibers in the skin connect with interneurons that may negatively modulate neural pathways to the pain centers in the cortex. This is the basis for the novel hypothesis for the involvement of purinergic signaling in acupuncture [46]. Furthermore, purines are known to cause intracellular Ca^2+^-dependent transient changes in cultured human fibroblast cytoarchitecture, which share similarities with the increase in cross-sectional area of fibroblasts in response to acupuncture [69]. Although evidence has been presented of the role of adenosine in acupuncture-mediated anti-nociception by demonstrating that the local concentrations of the nucleoside increase in human subjects [70] and that adenosine is implicated in the proliferation of fibroblasts and remodeling of the skin, liver and lung (reviewed in [37], we failed to demonstrate any contribution of the nucleoside to bradykinin-induced [Ca^2+^]_i_ signals in cultured human subcutaneous fibroblasts. Controversy still exists regarding adenosine participation in wound healing and scarring. For instance, the adenosine A_2A_ receptor promotes skin fibrosis and scarring [71] and increases collagen production in human dermal fibroblasts [72] probably by activating the G_s_/cyclicAMP pathway [73], yet it remains controversial how A_2A_ receptors increase collagen production since cyclicAMP has been found to decrease the synthesis of collagen and DNA by fibroblasts [74]. Thus, further studies are required to test long-term effects of extracellular adenosine, which may originate from the hydrolysis of ATP released from fibroblasts stimulated either mechanically (e.g. acupuncture) or by inflammatory mediators, such as bradykinin. Our findings demonstrating that adenosine accumulates as an end product of the catabolism of released ATP in the vicinity of fibroblasts within the subcutaneous connective tissue may be of clinical relevance, given the role of the nucleoside on dermal fibrosis (via A_2A_ receptors) and its anti-nociceptive properties (via A_1_ receptors) on free nerve endings and sensory afferents (reviewed in [37,75]).

## Conclusions

Data indicate that stimulation of constitutively expressed B_2_ receptors with bradykinin elicits the opening of hemichannels containing Cx43 and Panx1 subunits, followed by ATP diffusion out of human subcutaneous fibroblasts. ADP originating from the catabolism of released ATP by membrane-bound ectonucleotidases acting in an autocrine or paracrine way on plasma membrane P2Y_12_ purinoceptors may contribute to sustain elevated [Ca^2+^]_i_ levels in neighboring cells. Thus, targeting the pathways leading to nucleotides release and the purinergic cascade in human fibroblasts of the subcutaneous tissue may be useful in designing novel therapeutic strategies for tuning the communication between inflammatory cells, fibroblasts and sensory nerve endings, which are key players in the pathogenesis of painful musculoskeletal diseases with widespread involvement of the subcutaneous connective tissue (*e.g.* fibromyalgia).

## Methods

### Cell cultures

Human fibroblasts were isolated from the subcutaneous tissue of organ donors (51 ± 6 years old (mean ± S.E.M.), n = 13) with no clinical history of connective tissue disorders. The protocol was approved by the Ethics Committee of Centro Hospitalar do Porto (University Hospital) and of Instituto de Ciências Biomédicas de Abel Salazar (Medical School) of University of Porto (approval No. 11-CES-05). The investigation conforms to the principles outlined in the Declaration of Helsinki. Subcutaneous tissues were maintained at 4-6°C in M-400 transplantation solution (4.190 g/100 mL mannitol, 0.205 g/100 mL KH_2_PO_4_, 0.970 g/100 mL K_2_HPO_4_ · 3H_2_O, 0.112 g/100 mL KCl, and 0.084 g/100 mL NaHCO_3_, pH 7.4) until used, which was between 2 and 16 hours after being harvested [76]. Cells were then obtained by the explant technique and cultured in DMEM medium supplemented with 10% fetal bovine serum (FBS), 2.5 μg/mL of amphotericin B and 100 U/mL of penicillin/streptomycin, at 37°C in a humidified atmosphere of 95% air and 5% CO_2_. Medium was replaced twice a week. Primary cultures were maintained for 3-4 weeks, until near confluence, when adherent cells were enzymatically released with 0.04% trypsin-EDTA solution plus 0.025% type I collagenase in phosphate-buffered saline (PBS), at pH 7.4 during 15-20 minutes. The resultant cell suspension was cultured and maintained in the same conditions mentioned above. All the experiments were performed in the first subculture.

### Intracellular ATP imaging by quinacrine staining

Quinacrine is a weak-base acridine derivative that binds ATP with high affinity and specificity. When excited by light at 476 nm it emits fluorescence in the 500-540 nm range and it has been widely used to visualize ATP-containing sub-cellular compartments in living cells [77,78]. Human fibroblasts were seeded on 35 mm glass bottom dishes (FluoroDish®, World Precision Instruments), at a density of 2x10^4^ cells/mL, and maintained in culture for 5-15 days. To visualize ATP-filled vesicles, dishes were washed twice with Tyrode’s solution (137 mM NaCl, 2.7 mM KCl, 1.8 mM CaCl_2_, 1 mM MgCl_2_, 0.4 mM NaH_2_PO_4_, 11.9 mM NaHCO_3_, and 11.2 mM glucose, pH 7.4) before incubation with 30 μM quinacrine for 1 h [78]; cells were washed again twice and mounted onto a thermostatic (32°C) perfusion chamber at the stage of an inverted laser-scanning confocal microscope (Olympus FluoView^TM^ FV1000) equipped with a 40x magnification oil immersion objective lens (UPLAPO 40x OI; NA: 1.00). The chamber was continuously superfused (1 mL/min) with gassed (95% O_2_ + 5% CO_2_, pH 7.4) Tyrode’s solution and the cells were immediately observed. Changes in quinacrine fluorescence were detected in the time-lapse mode with FluoView Advanced Software (Olympus). Quinacrine was excited with a 488 nm Multi-line Ar laser and the emitted fluorescence was detected at 500-550 nm, using the scanner of the confocal microscope. Drugs were delivered using a multichannel perfusion system (ValveLink 8.2, Digitimer). Time-lapse sequences were recorded at scanning rates of 3 seconds per image, every 5 seconds, for about 30 minutes, digitized, and processed off-line by the computer. Regions of interest were defined as bright areas with as little background as possible.

### Extracellular ATP quantification by bioluminescence

Extracellular ATP was detected with the luciferin-luciferase ATP bioluminescence assay kit HS II (Roche Applied Science) using a Turner BioSystems (TD 20/20^n^) luminometer that accommodates 35-mm culture dishes, as described elsewhere [79]. Briefly, cells were seeded on 35 mm glass bottom dishes, at a density of 2x10^4^ cells/mL, for 5-15 days. At the beginning of the experiment, cells were washed twice with Tyrode’s solution at room temperature, before placing the dish in the luminometer reaction chamber. Bradykinin (30 μM) was added manually to the incubation fluid when bioluminescence baseline values reached stability after application of the luciferin/luciferase reagent. Experiments were performed at room temperature. ATP-dependent changes in extracellular luciferase activity were measured as relative luminescence unit (RLU) values integrated over 10 s photon counting periods.

### Measurement of intracellular Ca^2+^

Changes in the intracellular Ca^2+^ concentration ([Ca^2+^]_i_) were measured with the calcium sensitive dye Fluo-4 NW (Fluo-4 NW calcium assay kit, Molecular Probes, Invitrogen) in a multi detection microplate reader (Synergy™ HT Multi-Mode Microplate Reader, BioTek Instruments) [80]. In some of the experiments, single-cell [Ca^2+^]_i_ imaging was obtained using a laser scanning confocal microscope [22]. Briefly, human fibroblasts were seeded in flat bottom 96 well plates (Costar®, Corning® Inc.) at a density of 3x10^4^ cells/mL for the experiments using the microplate reader, and into 35 mm glass bottom dishes at a density of 2x10^4^ cells/mL for single-cell [Ca^2+^]_i_ imaging. Cells were cultured for 5-15 days in supplemented DMEM as described before. On the day of the experiment, cells were washed twice with Tyrode’s solution and incubated at 37°C for 45 min with the cell-permeant fluorescent Ca^2+^ indicator, Fluo-4 NW (2.5 μM), in 1X HBSS, 20 mM HEPES and 2.5 mM probenecid. After removal of the fluorophore loading solution, cells were washed again twice and 150/300 μL of Tyrode’s solution was added per culture well/dish, respectively. The 96-well cell plates were then loaded into the microplate reader. Reader control was performed using the BioTek’s Gen5™ Data Analysis Software. For the recordings, temperature was maintained at 32°C and readings were made with 5 seconds of interval, during approximately 30 minutes, using a tungsten halogen lamp. Fluorescence was excited at 485/20 nm and emission was measured at 528/20 nm. For the single-cell imaging, culture dishes were mounted on a thermostatic (32°C) perfusion chamber at the stage of an inverted laser-scanning confocal microscope equipped with a 20x magnification objective lens (LUCPLFLN 20x PH; NA: 0.45). The chamber was continuously superfused (1 mL/min) with gassed (95% O_2_, 5% CO_2_, pH 7.4) Tyrode’s solution and drugs were delivered using a multichannel valve controlled perfusion system. Changes in fluorescence of the Fluo-4 NW dye were detected in a time-lapse mode. Fluo-4 NW was excited with a 488 nm Multi-line Ar laser and the emitted fluorescence was detected at 510-560 nm, using the scanner of the confocal microscope. Time-lapse sequences were recorded at scanning rates with 20 seconds of interval for approximately 30 min, digitized, and processed off-line by the computer. Regions of interest were defined as bright areas with as little background as possible. For both methods, calcium measurements were calibrated to the maximal calcium load produced by ionomycin (5 μM, 100% response). In some experiments, we used rat subcutaneous fibroblasts obtained using the method described before to monitor single-cell [Ca^2+^]_i_ oscillations (see above) in parallel with the incorporation of FM4-64 (Molecular Probes, Invitrogen), which is a membrane-selective fluorescent dye used to evaluate vesicle endocytosis and exocytosis in living cells [33,34]. FM4-64 was excited with a 488 nm Multi-line Ar laser and fluorescence emission was detected at 665-765 nm, using the scanner of the confocal microscope. Drug application and time-lapse sequences were performed as above.

### Enzymatic kinetic experiments and HPLC analysis

The kinetics of ATP, ADP and AMP hydrolysis in human fibroblast cell cultures was evaluated at day 11, at 37°C. After a 30-min equilibration period, cells were incubated with 3 μM ATP, ADP or AMP, which were added to the culture medium at zero time. Samples (75 μl) were collected from each well at different times up to 30 min for high-performance liquid chromatography (HPLC, LaChrome Elite, Merck) analysis of the variation of substrate disappearance and product formation [22,76]. Concentrations of the substrate and products were plotted as a function of time (progress curves). The following parameters were analyzed for each progress curve: half-life time (t_1/2_) of the initial substrate, time of appearance of the different concentrations of the products, concentration of the substrate or any product remaining at the end of the experiment.

### Immunocytochemistry

Human subcutaneous fibroblasts were seeded in chamber slides at a density of 2.5x10^4^ cells/mL and allowed to grow for 5-15 days. Cultured cells were fixed in 4% paraformaldehyde (PFA) in PBS for 10 minutes, washed 3 times in PBS (10 minutes each) and, subsequently, incubated with blocking buffer I (10% FBS, 1% bovine serum albumin (BSA), 0.1% Triton-X, 0.05% NaN_3_) for 1 h. Primary antibodies, diluted in blocking buffer II (5% FBS, 1% BSA, 0.1% Triton-X, 0.05% NaN_3_), were applied [mouse anti-porcine vimentin 1:75 (DAKO); rabbit anti-human collagen I 1:50 (AbDSerotec); mouse anti-human α-smooth muscle actin-FITC 1:250 (Sigma-Aldrich); rabbit anti-human P2Y_12_ 1:100 (Alomone); rabbit anti-human Cx43 1:600 and rabbit anti-human Panx1 1:1000 (Abcam)] and the slides incubated overnight at 4°C. After incubation, cells were washed 3 times in PBS 1X (10 minutes each). The Alexa Fluor 488 1:1500 (anti-rabbit) and Alexa Fluor 568 1:1500 (anti-mouse) secondary antibodies (Molecular Probes, Invitrogen) were diluted in blocking buffer II (5% FBS, 1% BSA, 0.1% Triton-X) and applied for 1 h protected from light. A last wash was performed with PBS 1X and glass slides were mounted with VectaShield medium and stored at 4°C. For negative controls, the secondary antibodies were applied without pre-incubation with primary antibodies. A positive control for α-smooth muscle actin (SMA) was performed with cardiac myofibroblasts isolated from Wistar rats (Charles River, Barcelona) using a similar procedure as previously described for human subcutaneous fibroblasts. Observations were performed and analyzed with a laser-scanning confocal microscope [22].

### SDS–PAGE and Western blotting

Fibroblasts were homogenized in a lysis buffer with the following composition: 50 mM Tris-HCl (pH 8.0), 150 mM NaCl, 0.5% sodium deoxycholate, 1% Triton-X-100, 0.1% SDS and a protease inhibitor cocktail. Protein content of the samples was evaluated using the BCA protein assay kit according to the manufacturer’s instructions (Pierce). Samples were solubilized in SDS reducing buffer (0.125 mM Tris-HCl, 4% SDS, 0.004% bromphenol blue, 20% glycerol, and 10% 2-mercaptoethanol, pH 6.8 at 70°C for 10 min), subjected to electrophoresis in 10% SDS-polyacrylamide gels and electrotransferred onto PVDF membranes (MilliPore). Protein loads were 25 μg for Panx1 and 15 μg for Cx43. The membranes were blocked for 1 h in Tris-buffered saline (TBS: 10 mM Tris-HCl, pH 7.5, 150 mM NaCl) containing 0.05% Tween 20 + 5% BSA. Membranes were subsequently incubated with rabbit anti-human Panx1 1:250 (Novex, Life Technologies) and rabbit anti-human Cx43 1:6000 (Abcam) in the above blocking buffer overnight at 4ºC. Membranes were washed three times for 10 min in 0.1% Tween 20 in TBS and then incubated with donkey anti-rabbit IgG (HRP) 1:30000 (Abcam) secondary antibody, for 60 min at room temperature. For comparison purpose, the membranes were also incubated with rabbit anti-human β-tubulin 1:2500 (Abcam) antibody following the procedures described above. Membranes were washed three times for 10 min and antigen-antibody complexes were visualized by chemiluminescence with an ECL reagent using the ChemiDoc MP imaging system (Bio-Rad Laboratories).

### Reagents and materials

2-Mercaptoethanol, 2-octanol, 8-phenyltheophylline (8-PT), adenosine 5’-diphosphate sodium salt (ADP), adenosine 5’-monophosphate sodium salt (AMP), adenosine 5’-triphosphate sodium salt (ATP), amphotericin B, apyrase, bovine serum albumin (BSA), bradykinin (BK), brefeldin A (BFA), bromphenol blue, carbenoxolone (CBX), Dulbecco’s Modified Eagle’s Medium (DMEM), ethylene diaminetetraacetic acid (EDTA), ethylene glycol-bis(2-aminoethylether)-N,N,N’,N’-tetraacetic acid (EGTA), fetal bovine serum (FBS), glycerol, mefloquine (MFQ), 5’-(N-ethylcarboxamide)adenosine (NECA), penicillin/streptomycin, phosphate buffered saline system (PBS), protease inhibitor cocktail, quinacrine dihydrochloride, sodium deoxycholate, sodium dodecyl sulfate (SDS), trypsin, Tween 20, and type I collagenase were purchased from Sigma-Aldrich. ^10^Panx, 2-(propylthio)adenosine-5’-O-(β,γ-difluoro methylene) triphosphate tetrasodium salt (AR-C 66096), 2-[(2-chloro-5-nitrophenyl)azo]-5-hydroxy-6-methyl-3-[(phosphonooxy)methyl]-4-pyridine carboxaldehyde disodium salt (MRS 2211), 2’-deoxy-N6-methyladenosine 3’,5’-bisphosphate tetrasodium salt (MRS 2179), icatibant (HOE-140), R715, sodium metatungstate (POM-1) and thapsigargin were obtained from Tocris Cookson Inc.. Bafilomycin A1 was from WAKO Chemicals and Triton™ X-100 was obtained from Merck. BFA, MFQ, NECA and thapsigargin were prepared in dimethyl sulfoxide (DMSO). All other drugs were prepared in distilled water. All stock solutions were stored as frozen aliquots at -20°C. Dilutions of these stock solutions were made daily and appropriate solvent controls were done. No statistically significant differences between control experiments, made in the absence or in the presence of DMSO at the maximal concentration used (0.05% v/v), were observed. The pH of the solutions did not change by the addition of the drugs in the maximum concentrations applied to the preparations.

### Presentation of data and statistical analysis

Data are expressed as mean ± S.E.M. from an *n* number of experiments/cells/individuals. Data from different individuals were evaluated by one-way analysis of variance (ANOVA) and no significant differences on the pattern of cell behavior were found. Statistical differences found between control and drug-treated cultures were determined by the Bonferroni’s method. *p* values < 0.05 were considered to represent significant differences.

## Abbreviations

[Ca2+]i: Intracellular calcium; 8-PT: 8-phenyltheophylline; a.u.: Arbitrary units; ADA: Adenosine deaminase; ADO: Adenosine; AR-C 66096: 2-(propylthio)adenosine-5’-O-(β,γ-difluoro methylene) triphosphate tetrasodium salt; Baf A1: Bafilomycin A1; BCA: Bicinchoninic acid; BFA: Brefeldin A; BK: Bradykinin; BSA: Bovine serum albumin; CBX: Carbenoxolone; CD39: NTPDase1; CD73: Ecto-5’-nucleotidase; COS-7: Fibroblast-like cell line derived from monkey kidney tissue; Cx: Connexin; DMEM: Dulbecco’s modified eagle’s medium; DMSO: Dimethyl sulfoxide; DRG: Dorsal root ganglia; EDTA: Ethylene diaminetetraacetic acid; EGTA: Ethylene glycol-bis(2-aminoethylether)-N,N,N’,N’-tetraacetic acid; FBS: Fetal bovine serum; FITC: Fluorescein isothiocyanate; HBSS: Hank’s balanced salt solution; HEPES: 4-(2-hydroxyethyl)-1-piperazineethanesulfonic acid; HEK-293: Human embryonic kidney 293 cells; HPLC: High-performance liquid chromatography; HRP: Horseradish peroxidase; HX: Hypoxanthine; INO: Inosine; IP3: Inositol trisphophate; MDLK: Madin-Darby canine kidney cells; MFQ: Mefloquine; MRS 2179: 2’-deoxy-N6-methyladenosine 3’,5’-bisphosphate tetrasodium salt; MRS 2211: 2-[(2-chloro-5-nitrophenyl)azo]-5-hydroxy-6-methyl-3-[(phosphonooxy)methyl]-4-pyridine carboxaldehyde disodium salt; MTT: Thiazolyl blue formazan, 1-(4,5-dimethylthiazol-2-yl)-3,5-diphenylformazan; NECA: 5’-(N-ethylcarboxamide) adenosine; NTPDase: Nucleoside triphosphate diphosphohydrolase; Panx1: Pannexin-1; PBS: Phosphate buffered saline system; PFA: Paraformaldehyde; POM-1: Sodium metatungstate; PVDF: Polyvinyl difluoride; RLU: Relative luminescence units; SDS: Sodium dodecyl sulfate; SMA: α-smooth muscle actin; TBS: Tris-buffered saline; VSOR: Volume-sensitive outwardly rectifying; WB: Western blot.

## Competing interests

The authors declare that they have no competing financial interests.

## Authors’ contributions

All authors contributed to experimental design, execution of lab work and preparation of the manuscript. All authors read and approved the final manuscript.
